# *β*-Defensin Strengthens Antimicrobial Peritoneal Mast Cell Response

**DOI:** 10.1155/2020/5230172

**Published:** 2020-04-27

**Authors:** Justyna Agier, Ewa Brzezińska-Błaszczyk, Sylwia Różalska, Magdalena Wiktorska, Sebastian Wawrocki, Paulina Żelechowska

**Affiliations:** ^1^Department of Experimental Immunology, Faculty of Health Sciences, Medical University of Lodz, Lodz, Poland; ^2^Department of Industrial Microbiology and Biotechnology, Faculty of Biology and Environmental Protection, University of Lodz, Lodz, Poland; ^3^Department of Molecular Cell Mechanisms, Faculty of Health Sciences, Medical University of Lodz, Lodz, Poland; ^4^Department of Immunology and Infectious Biology, Faculty of Biology and Environmental Protection, University of Lodz, Lodz, Poland

## Abstract

Mast cells (MCs) are engaged in the processes of host defense, primarily *via* the presence of receptors responsible for the detection of pathogen-associated molecular patterns (PAMPs). Since BDs are exclusively host defense molecules, and MCs can elicit the antimicrobial response, this study is aimed at determining whether BDs might be involved in MC pathogen defense. We found that defensin BD-2 significantly augments the mRNA and protein expression of Toll*-*like receptors (TLRs) and retinoic acid-inducible gene-I-like receptor (RLR) essential for the detection of viral molecules, i.e., TLR3, TLR7, TLR9, and RIG-I in mature tissue rat peritoneal MCs (PMCs). We established that BD-2 might stimulate PMCs to release proinflammatory and immunoregulatory mediators and to induce a migratory response. Presented data on IgE-coated PMC upon BD-2 treatment suggest that in the case of allergies, there is an enhanced MC immune response and cell influx to the site of the ongoing infection. In conclusion, our data highlight that BD-2 might strongly influence MC features and activity, mainly by strengthening their role in the inflammatory mechanisms and controlling the activity of cells participating in antimicrobial processes.

## 1. Introduction

One of the critical components of the innate immune response is a family of small, multifunctional molecules known as antimicrobial peptides (AMPs). Most of AMPs are produced due to stimulation; however, most of them are generated incessantly by distinctive cells. The AMP family consists of two primary groups, cathelicidins and defensins, divided into three distinct subgroups—*α*, *β*, and *θ* [1, 2]. The *β*-defensins (BDs) are expressed by the skin and mucosal epithelial cell lining, i.e., the urinary tract, kidney, or trachea. Moreover, they can be expressed in monocytes, macrophages, and dendritic cells. The BDs have been reported to function as AMPs for the Gram-negative and Gram-positive bacteria, viruses, fungi, and even parasites by interacting with the membrane of the pathogen, thus, inducing its permeabilization [3, 4]. While BDs mainly serve as potent microbicidal agents, they also exhibit immunomodulatory properties. They appear to be essential chemokines, inducing the production and release of diverse immunoregulatory mediators by inflammatory cells; therefore, they are distinguished as active agents in inflammatory processes. BDs modulate mechanisms of adaptive immunity, and they supply a link between the innate and adaptive immune response as they can recruit immature memory T cells and dendritic cells to sites of infection and/or inflammation [5, 6].

Mast cells (MCs) have been principally viewed as effectors of allergic reactions [7], but now they are also considered to be fundamental in many other physiological and pathological processes [8, 9]. MCs are primarily found at the strategic sites that are susceptible to the external environment, close to blood vessels, lymphatic vessels, and a multitude of immune cells [10]. They are morphologically characterized by numerous large granules that store a plethora of biologically active preformed mediators such as histamine, proteoglycans, and metalloproteinases. MCs also have the capability of secreting *de novo*-generated arachidonic acid metabolites, as well as many newly synthesized cytokines and chemokines [11, 12]. Hence, MCs determine the chief sentinels of the immune system, with a plurality of functions in the maintenance of a range of physiological features. They are recognized as crucial for the control of body homeostasis by acting on angiogenesis, wound healing, and vascular permeability [8, 9]. Furthermore, MCs greatly influence both innate and acquired immune mechanisms [13]. Another noteworthy MC activity is to modulate acute and chronic inflammation, as well as play a vital role in orchestrating inflammatory response [14–16].

Because of their location at host-environment interfaces, it has been hypothesized that MCs can function as an orchestra conductor of defense toward invading microorganisms [17, 18]. In further support for this, MCs express a wide variety of surface and intracellular molecules known as pathogen-recognition receptors (PRRs), which recognize unique microbial components [19]. It is also well known that MCs exert several direct and indirect mechanisms of pathogen destruction, i.e., generation of various pro- and anti-inflammatory humoral factors, phagocytosis, extracellular trap formation, and antigen presenting through class I and II molecules of major histocompatibility complex (MHC) [17, 18].

Since BDs are exclusively host defense molecules, and MCs can elicit the antimicrobial response, this study is aimed at determining whether BDs might be involved in MC pathogen defense. The present research focuses on the examination of one selected AMP–BD-2 on the expression of receptors essential for the recognition of viral-associated molecular patterns, i.e., Toll-like receptors (TLRs) TLR3, TLR7, and TLR9 and retinoic acid-inducible gene-I-like receptors (RLRs) RIG-I in peritoneal MCs (PMCs). Furthermore, we also established the effects of BD-2 on PMC inflammatory activity, including cytokine/chemokine production and cell migration. Considering the MC's key role in allergic reactions, we also studied the effect of BD-2 on the Fc*ε*RI-dependent histamine release and cell migration.

## 2. Materials and Methods

### 2.1. Animals

The study was performed on female albino Wistar rats Crl:WI (Charles River Laboratories, Wilmington, MA, USA) weighing ~250 g, aged three to four months. The animals were obtained from the animal quarters of the Faculty of Biology and Environmental Protection of the University of Lodz. The experimental protocols were approved by the Local Ethics Committee for Experiments on Animals in Lodz (the approval No. 39/ŁB100/2018). All efforts were made to minimize animal suffering. All animals were treated with isoflurane-induced (Baxter, Deerfield, IL, USA) anesthesia before decapitation.

### 2.2. Isolation of PMCs

Peritoneal cell suspensions were obtained from peritoneal cavities by lavage with 50 mL of 1% HBSS (Gibco, Gaithersburg, MD, USA) supplemented with 0.015% sodium bicarbonate (Gibco). After abdominal massage (90 s), the cell suspension was removed from the peritoneal cavity. The peritoneal cell suspension was washed twice (150 x *g*, 5 min, and 20°C) in complete (c)DMEM containing DMEM (Biowest, Kansas City, MO, USA), supplemented with 10% FCS (GIBCO), 10 *μ*g/mL gentamicin (Gibco), and 2 mM glutamine (Gibco). Isotonic 72.5% Percoll (Sigma-Aldrich, St. Louis, MO, USA) density gradient centrifugation (190 x *g*, 15 min, and 20°C) was used for PMC purification. Subsequently, isolated PMCs were centrifuged twice in cDMEM (150 x *g*, 5 min, and 20°C). The isolation of PMCs lasted approximately 45-50 min. After being washed, PMCs were counted and resuspended in an appropriate volume of cDMEM (for quantitative RT-PCR, flow cytometry and confocal microscopy analysis, and migration assay) or medium for rat PMCs, containing 137 mM NaCl (Sigma-Aldrich), 2.7 mM KCl (Sigma-Aldrich), 1 mM MgCl_2_ (Sigma-Aldrich), 1 mM CaCl_2_ (Sigma-Aldrich), 10 mM HEPES (Sigma-Aldrich), 5.6 mM glucose (Sigma-Aldrich), and 1 mg/mL bovine serum albumin (BSA) (Sigma-Aldrich) (for histamine release assay, cytokine/chemokine release measurements, and ROS generation), to obtain PMC concentration of 1.5 × 10^6^ cells/mL. To acquire appropriate PMC density and the number of samples in a given type of experiment, a proper number of animals were used. MCs were prepared with purity > 98%, as determined by metachromatic staining with toluidine blue (Sigma-Aldrich). The viability of PMCs was over 98%, as determined by the trypan blue (Sigma-Aldrich) exclusion assay. The results of the treated samples were compared to the control from a given experiment.

### 2.3. Quantitative RT-PCR

qRT-PCR was used to determine BD-2-induced receptor and cytokine/chemokine mRNA levels in PMCs. Purified PMCs suspended in cDMEM were stimulated with BD-2 (PeptaNova GmbH, Sandhausen, DE) at a final concentration of 1 *μ*g/mL for 1, 2, or 3 h at 37°C in a humidified atmosphere with 5% CO_2_. For control, PMCs were incubated under the same conditions without BD-2. Total RNA was isolated from cells using the RNeasy® Mini Kit (Qiagen, Valencia, CA, USA). Then, cDNA was synthesized according to the manufacturer's instructions of iScript™ cDNA Synthesis Kit (Bio-Rad Laboratories, Hercules, CA, USA). qRT-PCR was performed on the CFX96 Touch™ Real-Time PCR Detection System (Bio-Rad Laboratories) using iTaq™ Universal SYBR® Green Supermix (Bio-Rad Laboratories). PCR reaction volumes consisted of 5 *μ*L of iTaq™ Universal SYBR® Green Supermix, 1 *μ*L of cDNA, 2 *μ*L of primers (5 mM), and 2 *μ*L of PCR-grade water included in the kit. Cycling conditions were as follows: initial denaturation at 95°C for 3 min followed by 40 cycles of denaturation in 95°C for 10 s, annealing in 60°C for 10 s, and then extension in 72°C for 20 s. The fold changes of the tested samples were calculated by the Bio-Rad CFX Maestro™ Software, based on the ^*ΔΔ*Ct^ method. The expression of receptor and cytokine/chemokine mRNAs was corrected by normalization based on the transcript level of the housekeeping gene rat *Actb*. For the calibrator samples, nonnstimulated specimens were used. Primer sequences are shown in Table 1.

### 2.4. Cell Preparation for Flow Cytometric and Confocal Microscopy Analysis

Constitutive and BD-2-induced TLR3, TLR7, TLR9, and RIG-I expressions were determined using flow cytometry and confocal microscopy. Constitutive expression of TLR3, TLR7, TLR9, and RIG-I was assessed in native PMCs (nonstimulated cells). Induced receptor expression was estimated in PMCs incubated with BD-2, at a final concentration of 1 *μ*g/mL, for 1 or 3 h at 37°C in a humidified atmosphere with 5% CO_2_. Following this, the PMCs were fixed with CellFIX™ (BD Bioscience, San Jose, USA) solution for 15 min at 4°C and washed twice with 1x PBS (Cayman Chemical, Ann Arbor, USA). To determine the intracellular localization of receptors, the PMCs were permeabilized with 0.1% saponin (Sigma-Aldrich) for 30 min at room temperature. Next, PMCs were resuspended in 1x PBS and stained for 1 h with goat anti-TLR3, goat anti-TLR7, rabbit anti-TLR9, or goat anti-RIG-I antibodies (Santa Cruz Biotechnology, Inc., Dallas, USA) (dilution 1 : 100). For control, PMCs were stained with goat or rabbit IgG isotype control (R&D Systems, Minneapolis, USA) with irrelevant specificity. The primary antibody was not added to the sample to certify the nonspecific binding of the secondary antibody. Cells were then washed with 1x PBS and incubated with Alexa Fluor 488® rabbit anti-goat IgG or Alexa Fluor 488® goat anti-rabbit IgG (Jackson ImmunoResearch Laboratories, Inc., West Grove, USA) (dilution 1 : 100) in 1x PBS for 1 h in the dark. Following this, the cells were washed twice and finally resuspended in 1x PBS before receptor assessment. After each period of incubation, PMC viability was examined using the trypan blue exclusion test.

### 2.5. Flow Cytometry

Ten thousand events in each sample were analyzed using a FACSCalibur flow cytometer with CellQuest software (BD Biosciences). BD-2-dependent PMC TLR3, TLR7, TLR9, and RIG-I expressions were presented as a percentage of TLR3/TLR7/TLR9/RIG-I MFI (mean fluorescence intensity) measured in native PMCs (referred to as 100%).

### 2.6. Confocal Microscopy

The samples were mounted on microscope slides, and images were captured using a Leica TCS SP8 microscope (Wetzlar, Germany) with the HC PL APO CS2 63x/1.4 oil objective at the Laboratory of Microscopic Imaging and Specialized Biological Techniques University of Lodz. The 488 nm laser was used to excite the fluorescence, and the emission was collected by a hybrid detector in the range of 505–550 nm. To visualize the cells, the PMT transmission channel was used. LAS X 2.0.2.15022 software (Leica Microsystems, Wetzlar, Germany) was used to analyze the data. All settings were held constant throughout the experiments. All signals obtained from confocal microscopy were validated with profile view image analysis, and the diagrams presenting intensity values were placed under each microphotograph. The mean fluorescence intensity (expressed in arbitrary units (AU)) was calculated for each of the samples. The calculations were performed for at least 40 different points randomly selected in compartments with receptor expression.

### 2.7. ELISA

For cytokine and chemokine generation measurements, purified PMCs suspended in medium for rat PMCs were incubated with BD-2 at final concentrations of 1, 5, 10, 20, and 40 *μ*g/mL or buffer alone (spontaneous cytokine/chemokine generation) in a humidified atmosphere with 5% CO_2_ for 3 h at 37°C. The supernatants were collected by centrifugation. IFN-*α*, IFN-*β*, IFN-*γ*, GM-CSF, CCL2, and CCL3 concentrations in supernatants were evaluated by ELISA kits (IFN-*α*, IFN-*β*: Wuhan EIAab Science Inc., Wuhan, China; IFN-*γ*, GM-CSF, and CCL3: Biorbyt Ltd., Cambridge, UK; and CCL2: Cloud-Clone Corp., Katy, TX, USA) according to the manufacturer's instructions. The sensitivity of those tests was <31.2 pg/mL, <31.2 pg/mL, <7 pg/mL, <7 pg/mL, <1 pg/mL, and < 15 pg/mL, respectively.

### 2.8. Histamine Release Assay

Purified PMCs suspended in medium for rat PMCs were incubated with BD-2 at final concentrations of 1, 5, 10, 20, and 40 *μ*g/mL, compound 48/80 (Sigma-Aldrich), a commonly-known potent MC degranulation factor, at a final concentration of 5 *μ*g/mL (positive control) or buffer alone (spontaneous histamine release) in a water bath for 30 min at 37°C with constant stirring. In another series of experiments, PMCs were preincubated with rat purified IgE (Invitrogen, Carlsbad, CA, USA) at 1 *μ*g/mL for 1 h at 37°C with constant stirring. After preincubation, PMCs were washed in cDMEM (150 x *g*, 5 min, and 20°C) and next stimulated with BD-2 at final concentrations of 1, 5, 10, 20, and 40 *μ*g/mL. For time-course experiments, PMCs were incubated with BD-2 at a final concentration of 20 *μ*g/mL for 0, 1, 3, 5, 10, and 30 min. After incubation, the reaction was stopped by adding 1.9 mL of cold medium. Next, the cell suspension was centrifuged, and the supernatants were demounted into other tubes. A total of 2 mL distilled water was added to each tube with the cell pellets. Histamine content was determined in both cell pellets (residual histamine) and supernatants (released histamine) by the spectrofluorometric method, as previously described [20]. Histamine release was expressed as a percentage of the total cellular content of the amine.

### 2.9. Migration Assay

The PMC migratory response to BD-2 was examined *in vitro* using Boyden microchamber assay (Neuro Probe, Gaithersburg, USA) in a 48-well chemotaxis chamber (Neuro Probe). Thirty microliters of BD-2, at final concentrations of 1, 5, 10, 20, and 40 *μ*g/mL, tumor necrosis factor (TNF) (R&D Systems) at a final concentration of 0.05 pg/mL, CCL5 (R&D Systems) at a final concentration of 100 ng/mL, nerve growth factor (NGF) (R&D Systems) at a final concentration of 100 ng/mL, LL-37 (AnaSpec, Fremont, CA, USA) at a final concentration of 20 *μ*g/mL, and CRAMP (AnaSpec) at a final concentration of 20 *μ*g/mL or buffer alone (control spontaneous migration) was placed into the lower compartments of the microchamber. The lower compartments were covered with a polycarbonate 8 *μ*m-pore-size membrane, and 50 *μ*L of the cell suspensions was applied to the upper compartments. Purified PMCs were also preincubated with IgE at 1 *μ*g/mL for 1 h at 37°C with constant stirring. After preincubation, PMCs were washed in cDMEM (150 x *g*, 5 min, and 20°C). Next, appropriated BD-2 concentrations were placed into the lower compartments of a microchamber, and 50 *μ*L of IgE-coated PMCs was pipetted into the upper compartments. Subsequently, the chemotaxis chamber was incubated for 3 h in a humidified atmosphere with 5% CO_2_ at 37°C. After the incubation period, PMCs adherent to the upper surface of the membrane were removed by scraping with a rubber blade. Migrating cells adherent to the lower surface of the membrane were fixed in 99.8% ethanol, stained for 10 min with hematoxylin, cleared in distilled water, and mounted on a microscope slide. PMC migration was quantified by counting the number of cells that had traversed the membrane and were attached to the bottom surface of the filter. Ten high-power fields (HPF) were calculated in each assay (×250). Spontaneous migration served as a control and was referred to as 100%. The results were presented as a percentage of control migration.

### 2.10. Measurement of Intracellular ROS Production

For determination of ROS generation, PMCs suspended in the medium for rat PMCs were incubated with BD-2 at final concentrations of 1, 5, 10, 20, and 40 *μ*g/mL, or with medium alone for 1 h in a humidified atmosphere with 5% CO_2_ at 37°C. CellROX™ Green Reagent (Invitrogen) at a final concentration of 5 *μ*M was added and incubated with cells at 37°C for 30 min. The cells were then washed three times with 1x PBS and analyzed on FLUOstar Omega Microplate Reader (BMG LABTECH, Ortenberg, Germany). Fluorescence intensity was analyzed at the excitation/emission wavelengths of 485/520 nm. ROS generation was expressed as MSI (mean signal intensity).

### 2.11. Statistical Analysis

The statistical analysis of the experimental data was performed using Statistica 13 software (Statsoft Inc., USA). Data are presented as mean ± standard deviation (SD). Normality of distribution was tested with the Shapiro-Wilk test. All comparisons between groups were carried out by using Student's *t*-test for small groups. Differences were considered significant at *P* < 0.05 and are labeled with an asterisk (^∗^) on each graph.

## 3. Results

### 3.1. The Effect of BD-2 on TLR3, TLR7, TLR9, and RIG-I Expressions in PMCs

**Figure 1:**
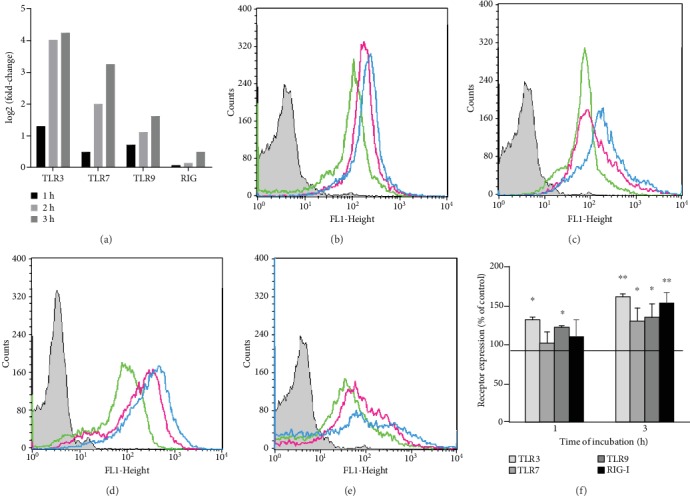
Constitutive and BD-2-induced TLR3, TLR7, TLR9, and RIG-I mRNA and protein expressions in PMCs. PMCs were incubated with medium alone (resting nonstimulated cells) or BD-2 at a final concentration of 1 *μ*g/mL, and TLR3, TLR7, TLR9, and RIG-I expressions were assessed by (a) qRT-PCR and (b–f) flow cytometry. Representative flow cytometry histogram showing (b) TLR3, (c) TLR7, (d) TLR9, and (e) RIG-I expressions. Shaded tracings—isotype control and open tracings—receptor expression in resting cells (green), and after BD-2 stimulation for 1 h (violet) and 3 h (blue). (f) The effect of BD-2 on TLR3/7/9/RIG-I expression on rat MCs. Constitutive receptor expression served as a control and was referred to as 100%. Results are the mean of fluorescent intensity ± SD of three experiments performed in duplicate. Differences were considered significant at *P* < 0.05 and are labeled with an asterisk (^∗^) on each graph (Student's *t*-test). ^∗^*P* < 0.05, ^∗∗^*P* < 0.01.

Firstly, we determined whether stimulation with BD-2 influences TLR3, TLR7, TLR9, and RIG-I mRNA expressions in mature rat PMCs. The qRT-PCR technique was used to assess the expression level of receptor mRNAs in resting PMCs and those stimulated with 1 *μ*g/mL BD-2. As demonstrated in Figure 1(a), defensin stimulation resulted in 16.5-, 3.9-, and 1.8-fold upregulation of TLR3, TLR7, and TLR9 mRNAs, respectively. BD-2 did not affect RIG-I mRNA expression.

Next, we checked the expression of TLR3, TLR7, TLR9, and RIG-I proteins in tissue PMCs, and we determined whether BD-2 has an impact on their baseline levels. Receptor expression was evaluated on nonstimulated PMCs, as well as on cells exposed to BD-2 at a concentration of 1 *μ*g/mL for 1 or 3 h. Flow cytometry confirmed the constitutive expression of intracellular TLR3, TLR7, TLR9, and RIG-I receptors (Figures 1(b)–1(e)). We found that the TLR3 expression level was significantly affected upon 1 h (*P* < 0.05) and 3 h (*P* < 0.01) stimulation, reaching 131.39 ± 10.18% and 160.65 ± 5.35% of control TLR3 expression in PMCs, respectively (Figure 1(f)). BD-2 enhanced TLR7 protein level, and the intensity of the signals was the highest after 3 h of stimulation (129.79 ± 18.28% of control TLR7 expression; *P* < 0.05). Moreover, 3 h PMC stimulation with BD-2 resulted in an increased TLR9 expression compared to nonstimulated cells (134.28 ± 16.53% of control TLR9 expression; *P* < 0.05). The RIG-I expression level was significantly affected upon 3 h stimulation, reaching 153.21 ± 18.27% of control RIG-I expression in PMCs.

**Figure 2:**
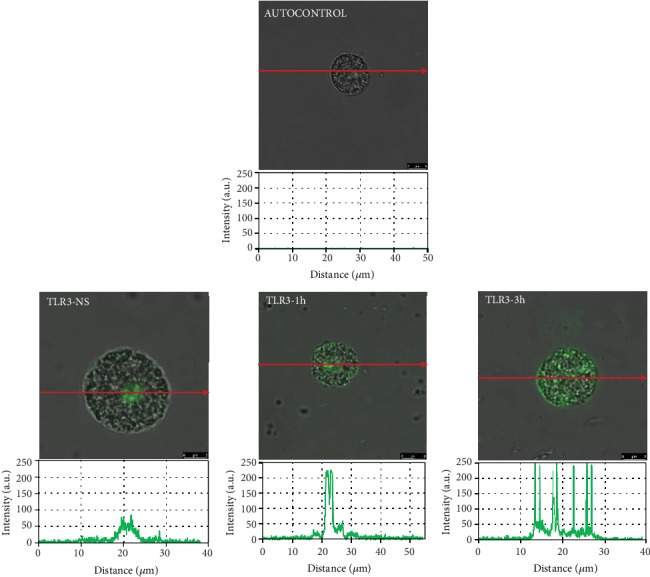
Effect of BD-2 stimulation on TLR3 expression in PMCs. PMCs were incubated with medium alone (NS: nonstimulated cells) or BD-2 for 1 or 3 h at a final concentration of 1 *μ*g/mL. Representative images showing TLR3 receptor cellular localization in permeabilized native, BD-2 stimulated for 1 h or BD-2 stimulated for 3 h PMCs analyzed by confocal microscopy. Single confocal sections (the midsection of cells) reveal the presence of receptors. Fluorescence intensity diagrams showing the distribution of fluorescence in cells were mounted.

**Figure 3:**
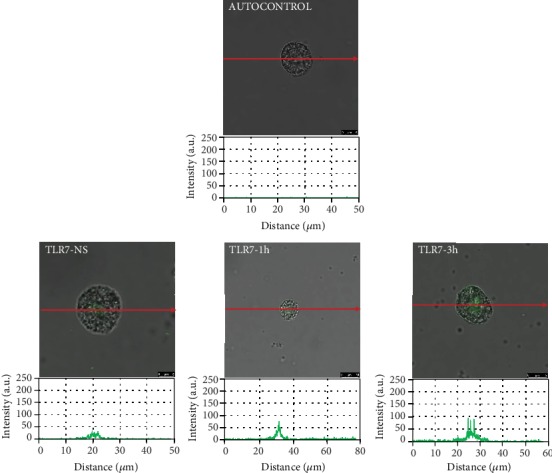
Effect of BD-2 stimulation on TLR7 expression in PMCs. PMCs were incubated with medium alone (NS: nonstimulated cells) or BD-2 for 1 or 3 h at a final concentration of 1 *μ*g/mL. Representative images showing TLR7 receptor cellular localization in permeabilized native, BD-2 stimulated for 1 h or BD-2 stimulated for 3 h PMCs analyzed by confocal microscopy. Single confocal sections (the midsection of cells) reveal the presence of receptors. Fluorescence intensity diagrams showing the distribution of fluorescence in cells were mounted.

**Figure 4:**
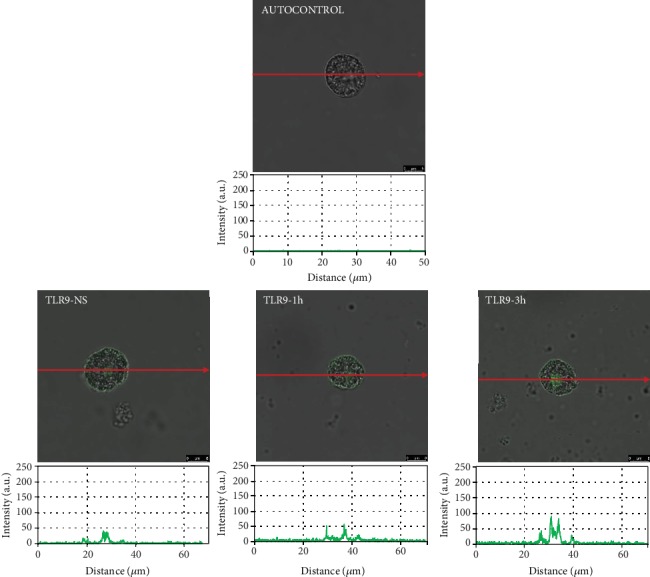
Effect of BD-2 stimulation on TLR9 expression in PMCs. PMCs were incubated with medium alone (NS: nonstimulated cells) or BD-2 for 1 or 3 h at a final concentration of 1 *μ*g/mL. Representative images showing TLR9 receptor cellular localization in permeabilized native, BD-2 stimulated for 1 h or BD-2 stimulated for 3 h PMCs analyzed by confocal microscopy. Single confocal sections (the midsection of cells) reveal the presence of receptors. Fluorescence intensity diagrams showing the distribution of fluorescence in cells were mounted.

**Figure 5:**
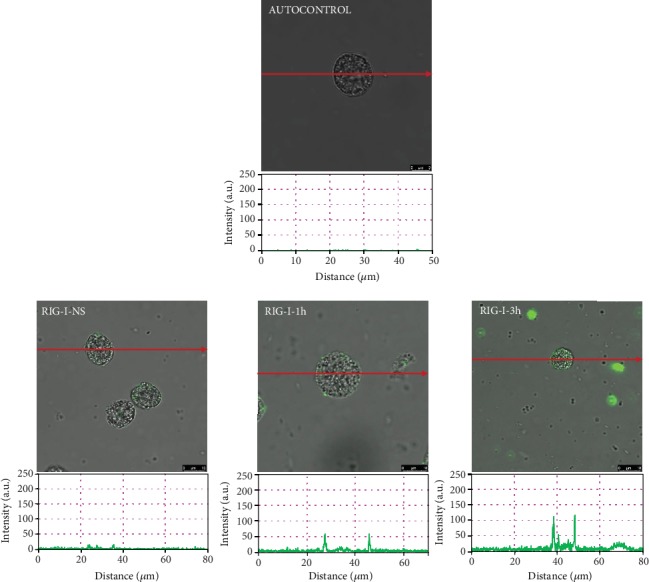
Effect of BD-2 stimulation on RIG-I expression in PMCs. PMCs were incubated with medium alone (NS: nonstimulated cells) or BD-2 for 1 or 3 h at a final concentration of 1 *μ*g/mL. Representative images showing RIG-I receptor cellular localization in permeabilized native, BD-2 stimulated for 1 h or BD-2 stimulated for 3 h PMCs analyzed by confocal microscopy. Single confocal sections (the midsection of cells) reveal the presence of receptors. Fluorescence intensity diagrams showing the distribution of fluorescence in cells were mounted.

To assess the location and distribution of TLRs and one RLR, a confocal microscopy technique was used. The changes in TLR3 expression are shown in Figure 2. The confocal microscopy and image analysis confirmed that in nonstimulated cells (NS), fluorescence is predominantly associated with the nuclear envelope, but this receptor is also located on the cell surface. PMC stimulation with BD-2 resulted in an increase of signals both in the cell membrane and intracellular regions after 1 h (215.30 ± 6.47 AU) and for an extended time (232.38 ± 18.38 AU) compared with NS cells. As demonstrated in Figure 3, in freshly isolated PMCs, TLR7 is localized mainly in the cell interior. BD-2-induced TLR7 expression increased in the cell interior, which was evidenced by fluorescence intensity diagrams. BD-2 sharply augmented expression of TLR7 after 3 h exposure (92.62 ± 3.31 AU). The changes in the TLR9 expression level are shown in Figure 4. In resting PMCs, TLR9 is predominantly found in the nucleus envelope and a weak signal was obtained from the cell surface. The TLR9 expression was significantly upregulated upon incubation with BD-2. Image analysis revealed that the intensity of cell surface fluorescence was considerably higher inside the cell at 1 h (46.49 ± 6.51 AU) and 3 h (79.18 ± 5.99 AU). As demonstrated in Figure 5, RIG-I is located not only in the intracellular space but also below the cell membrane of native cells. In the interior of nonstimulated permeabilized cells, a similar fluorescence signal was found to be associated with the cytoplasm and outer membrane. PMC treatment with BD-2 caused an enhancement of RIG-I near the cell surface after 1 h of incubation, which was confirmed by intensity diagram analysis. The intensity of the signals was statistically higher after 3 h (94.65 ± 10.42 AU) in comparison to NS cells.

### 3.2. The Effect of BD-2 on PMC Cytokine/Chemokine mRNA Levels

**Figure 6:**
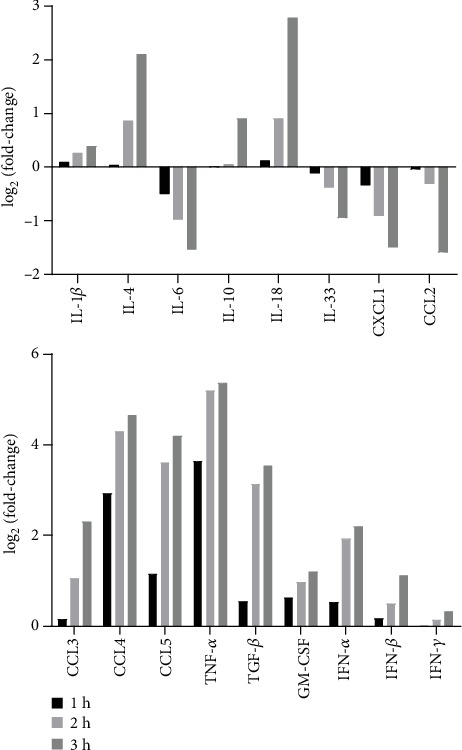
Effect of BD-2 on PMC cytokine/chemokine mRNA expression. PMCs were incubated with BD-2 (1 *μ*g/mL) for 1, 2, or 3 h. Total mRNA was extracted and converted into cDNA, and qRT-PCR was conducted to evaluate cytokine/chemokine mRNA expression. The expression of mRNAs was corrected by normalization based on the transcript level of the housekeeping gene rat *Actb*. Results are expressed as the mean of three independent experiments.

To establish whether BD-2 could provoke an inflammatory and immunoregulatory response by PMCs, we first evaluated the ability of this peptide to influence cytokine/chemokine mRNA expression. qRT-PCR was carried out, and the fold change of cytokine/chemokine mRNA expression in BD-2-stimulated PMCs (1 *μ*g/mL) compared to nonstimulated cells was assessed (Figure 6). Among the cytokines/chemokines measured in BD-2-stimulated PMCs, the highest mRNA expression levels were observed for proinflammatory ones, i.e., TNF (37.7-fold increase), CCL4 (20.2-fold increase), and CCL5 (12.5-fold increase). Also, the mRNA level for pleiotropic TGF-*β* was higher in BD-2-stimulated PMCs in comparison to nonstimulated cells (9.1-fold increase). Stimulation of PMCs with BD-2 also resulted in a substantial increase in the mRNA expression of IFN-*α* (3.9-fold), CCL3 (2.1-fold), GM-CSF (2-fold), IL-18 (1.8-fold), and IFN-*β* (1.5-fold). PMC incubation with BD-2 led to the reduced mRNA expression level of IL-6, CXCL1, and CCL2. At the same time, we documented that BD-2 did not influence IL-1*β*, IL-10, IL-33, and IFN-*γ* mRNA expressions in those cells.

### 3.3. The Effect of BD-2 on PMC Cytokine/Chemokine Generation

**Figure 7:**
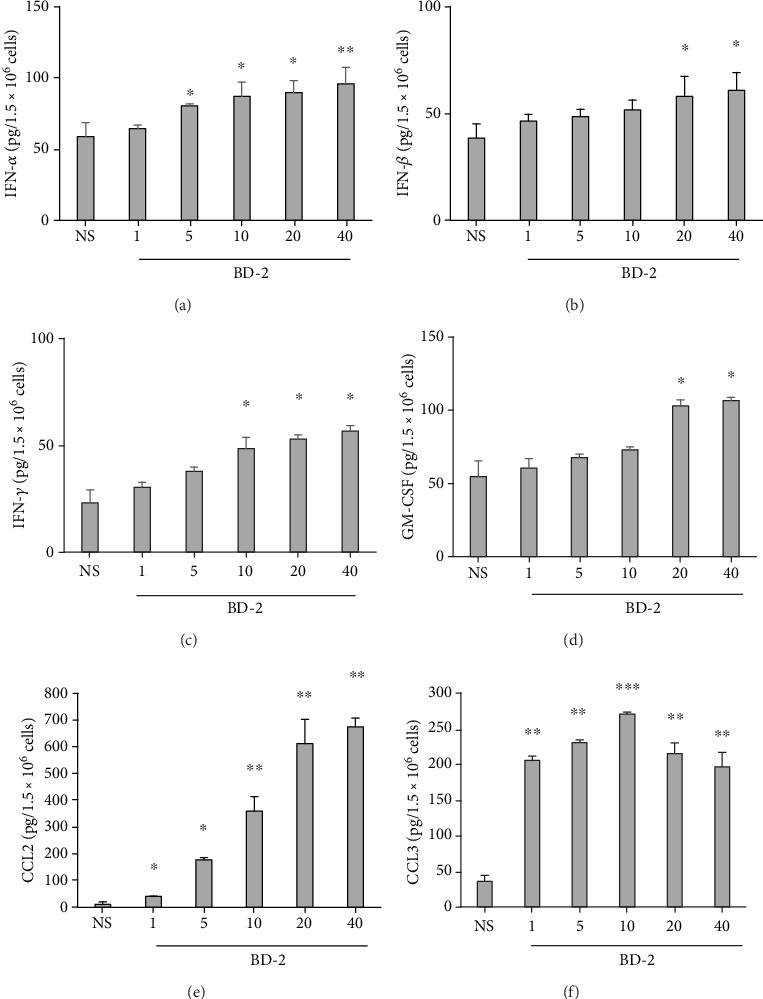
Effect of BD-2 on PMC (a) IFN-*α*, (b) IFN-*β*, (c) IFN-*γ*, (d) GM-CSF, (e) CCL2, and (f) CCL3 generation. PMCs were incubated with different concentrations of BD-2 or medium alone (NS) for 3 h. Results are the mean ± SD of three independent experiments done in duplicate. ^∗^*P* < 0.05, ^∗∗^*P* < 0.01, and ^∗∗∗^*P* < 0.001.

Next, we investigated whether BD-2, used at different concentrations, activates PMCs to IFN-*α*, IFN-*β*, and IFN-*γ* production. To this end, PMCs were stimulated with BD-2 at concentrations of 1, 5, 10, 20, and 40 *μ*g/mL for 3 h, using the medium alone as a negative control. After incubation, the levels of IFNs in supernatants were determined by specific ELISA kits. The results of those experiments are shown in Figures 7(a)–7(c). It was found that BD-2 induced all IFN release. Of the selected concentrations of BD-2, the most significant IFN-*α*, IFN-*β*, and IFN-*γ* secretions were observed at 40 *μ*g/mL, rising to 95.8 ± 4.2 pg per 1.5 × 10^6^ PMCs, 60.8 ± 8.30 pg per 1.5 × 10^6^ PMCs, and 56.6 ± 2.5 pg per 1.5 × 10^6^ PMCs, respectively.

Similarly, 20 *μ*g/mL and 40 *μ*g/mL BD-2 induced a considerable release of GM-CSF (*P* < 0.05) (Figure 7(d)). BD-2 at concentrations of 1, 5, 10, 20, and 40 *μ*g/mL was also found to be capable of triggering significant CCL2 and CCL3 production by PMCs (Figures 7(e) and 7(f)). CCL2 secretion in response to PMC stimulation with BD-2 used at 40 *μ*g/mL was the highest and up to 675.3 ± 17.30 pg per 1.5 × 10^6^ PMCs. In turn, when treated with 10 *μ*g/mL BD-2, CCL3 secretion by MCs peaked at 270.6 ± 14.48 pg per 1.5 × 10^6^ PMCs.

### 3.4. The Effect of BD-2 on Histamine Release

**Figure 8:**
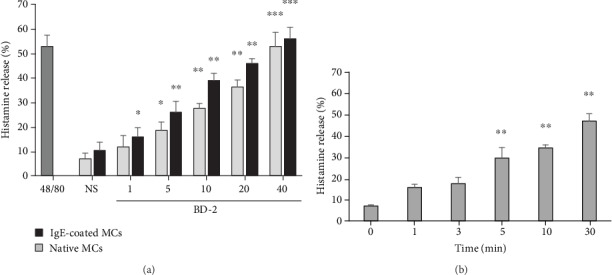
Effect of BD-2 on PMC histamine release. (a) Native PMCs or IgE-coated PMCs were incubated with different concentrations of BD-2, compound 48/80 at 5 *μg*/mL (positive control) or medium alone (NS) for 30 min. (b) Native PMCs were stimulated with BD-2 at 20 *μ*g/mL at the indicated time periods. Results are the mean ± SD of three independent experiments done in duplicate. ^∗^*P* < 0.05, ^∗∗^*P* < 0.01, and ^∗∗∗^*P* < 0.001.

The effect of various concentrations of BD-2 on native and IgE-coated PMC degranulation and histamine release was also evaluated. As demonstrated in Figure 8(a), BD-2 activated PMCs to a dose-dependent histamine release at 5-40 *μ*g/mL concentrations. PMC sensitization with IgE caused increased degranulation and histamine release in response to all BD-2 concentrations used. A potent degranulation inducer, i.e., compound 48/80, induced PMC histamine secretion up to 54.6 ± 4.8%. Time-course experiments revealed statistically significant (*P* < 0.01) histamine release within 5 min of incubation with BD-2 (Figure 8(b)).

### 3.5. The Effect of BD-2 on PMC Migration

**Figure 9:**
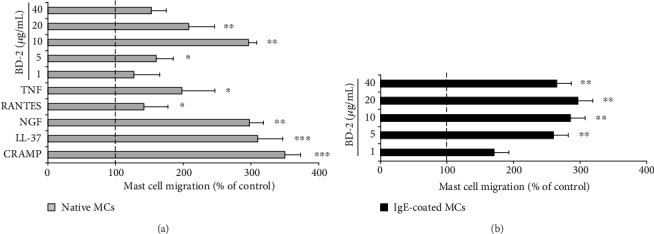
Effect of BD-2 on PMC migration. (a) Native PMCs or (b) IgE-coated PMCs were incubated with different concentrations of BD-2, TNF (0.05 pg/mL), CCL5 (100 ng/mL), NGF (100 ng/mL), LL-37 (20 *μ*g/mL), CRAMP (20 *μ*g/mL) or medium alone (control spontaneous PMC migration) for 3 h at 37°C in a Boyden microchamber. Spontaneous migration served as a control and was referred to 100%. Each point represents the mean ± SD of three independent experiments done in duplicate. ^∗^*P* < 0.05, ^∗∗^*P* < 0.01, and ^∗∗∗^*P* < 0.001.

Defensin BD-2 was checked for its chemotactic potency on native and IgE-coated PMCs for 3 h in a Boyden microchamber. The results were compared with those obtained for the migration of native PMCs in response to cytokines, known as potent MC chemoattractants, i.e., TNF, CCL5, and NGF, as well as in response to endogenous peptides such as human LL-37, and rat CRAMP. Results indicate that BD-2 at concentrations from 5 to 40 *μ*g/mL strongly induced migratory response of native PMCs, as compared to spontaneous migration (Figure 9(a)). We found that pretreatment of PMCs with 1 *μ*g/mL of IgE strongly enhanced BD-2-induced migration (Figure 9(b)).

### 3.6. The Effect of BD-2 on ROS Production by PMCs

**Figure 10:**
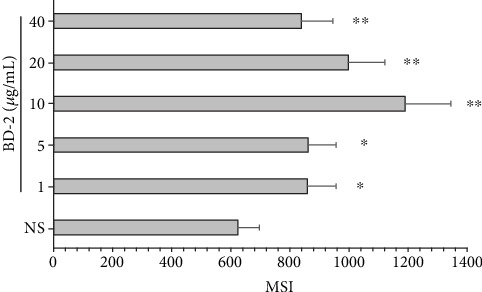
Effect of BD-2 on ROS generation. PMCs were incubated with different concentrations of BD-2 or medium alone (NS) for 60 min. CellROX™ Green Reagent was used at a concentration of 5 *μ*M for 30 min. Results are the mean signal intensity (MSI) ± SD of three independent experiments performed in duplicate (*n* = 6). ^∗^*P* < 0.05, ^∗∗^*P* < 0.01.

Spectrofluorimetry was used to examine ROS generation by PMCs in response to stimulation with BD-2. As demonstrated in Figure 10, PMCs produced significant amounts of ROS after stimulation with BD-2. Of the various concentrations of BD-2, the most significant ROS generation was observed at 10 *μ*g/mL, rising to 1190.3 ± 154.5 MSI.

## 4. Discussion

The multifunctional AMPs are essential for immune responses to infection. They are natural, broad-spectrum antimicrobials against both Gram-positive and Gram-negative bacteria, enveloped viruses, and fungi. Those small peptides kill the invaded pathogens directly by the disintegration of the microbial cell wall/membrane and/or lipid envelope and consequently leads to cell death. Moreover, AMPs participate in the neutralization of endotoxins and reengineer bacterial biofilms. Nowadays, more and more data indicate that cathelicidins and defensins, in addition to their antimicrobial features, possess various immunomodulatory activities. They have the potential to influence and modulate, both directly and indirectly, the activity of different cell populations involved in inflammatory processes and host defense against invading pathogens. Reported activities of AMPs include chemoattractant function, inhibition of neutrophil apoptosis, and ROS production. Also, those peptides directly activate inflammatory cells to the production and release of different proinflammatory and immunoregulatory mediators, cytokines, and chemokines. However, AMPs might mediate the generation of anti-inflammatory cytokines, as well [21–23].

To date, it is well established that MCs play a crucial immune surveillance role during infections and inflammation [15, 16]. Considering the data documenting the role of MCs in defense mechanisms and taking into account information on the significance of AMPs in these processes, it seems to be of importance finding whether those peptides modulate the antimicrobial activity of MCs. There are data that cathelicidins can directly activate MCs to proinflammatory activity. It was reported that human cathelicidin LL-37 induces MC degranulation [24–28], triggers LAD2 cells to LTC_4_ and PGE_2_ release [28], and leads to the production of cytokines, such as IL-1*β*, IL-2, IL-4, IL-5, IL-6, IL-31, TNF, GM-CSF, and chemokine CCL4 by those cells [24, 25, 27]. Hitherto, we stated that LL-37 stimulates rat MCs to histamine secretion and induces proinflammatory cytokine and chemokine mRNA/protein expression [29, 30]. We have also demonstrated that rat cathelicidin CRAMP induces cysLT generation and release, stimulates MCs to TNF synthesis, provokes GM-CSF, IL-1*β*, CCL2, and CCL3 mRNA expressions, and serves as a potent chemoattractant for MCs [31].

Although evidence suggests that cathelicidins can directly activate immune cells, only a handful of studies so far have investigated MC stimulation by defensins. Stimulation with BD-2, BD-3, and BD-4 caused PGD_2_, PGE_2_, and LTC_4_ production in rat MCs [28, 32, 33]. Niyonsaba et al. [28] observed that BD-1 to BD-4 induce IL-2, IL-4, IL-6, IL-8, and IL-31 release in the LAD2 immature human MC line. BDs cause MC degranulation, as demonstrated previously by histamine and *β*-hexosaminidase release assessment [28, 32, 33]. It has also been shown that BD-1 to BD-4 induce a migratory response in MCs [28, 32]. Whereas the impact of BDs on MCs is poorly understood, we decided to evaluate the influence of BD-2 on selected aspects of MC biology, in particular, those involved in antiviral defense.

In this study, we confirmed the expression of TLR3, TLR7, TLR9, and RIG-I mRNA and protein in matured *in vivo* rat MCs isolated from the peritoneal cavity, which are connective tissue-type MCs. However, it should be stressed that data relating to the AMP-induced modulation of their expression on MCs are scarce. Previously, Yoshioka et al. [34] evaluated the effect of LL-37 on TLR4 expression in MCs and noticed that this cathelicidin upregulates TLR4 expression in LAD2 cells. In turn, we stated that cathelicidin LL-37 influences TLR/RLR levels directly, enhancing TLR2, TLR4, and TLR9 expressions on the surface of MCs, and TLR3, TLR5, TLR7 [30], and RIG-I [35] in their interior. These findings led us to investigate whether another AMP, i.e., defensin BD-2, exert a similar effect on TLRs and RLRs. The result of the present study is that BD-2 may increase mainly the expression of TLR3 in rat PMCs that was analyzed by qRT-PCR, flow cytometry, and confocal microscopy analysis. It should be noted that TLR3 recognizes double-stranded RNA, genetic information carried by some viruses. Furthermore, we established that expression and distribution of other TLRs involved in identifying viral-associated molecular patterns, i.e., TLR7 and TLR9, are enhanced by defensin BD-2 in PMCs. The expression of other viral sensors RIG-I is also increased under the influence of BD-2. Those results confirm that BD-2 increases MC susceptibility to pathogen antigens, in particular viruses.

Our present findings indicate that BD-2 may also stimulate an antimicrobial response in rat PMCs. Upon interacting with BD-2, MCs release IFN-*α*, IFN-*β*, IFN-*γ*, GM-CSF, CCL2, and CCL3. Additionally, PMC activation with BD-2 led to dose-dependent and time-dependent PMC degranulation, as revealed by histamine release assessment. It is worthy to note that BD-2 sharply increases the mRNA expression of the cytokines and chemokines, i.e., IFN-*α*, IFN-*β*, TNF, TGF-*β*, GM-CSF, IL-18, CCL4, CCL5, and CCL3. These observations support the proposed role of BD-2 as endogenous amplifiers of the proinflammatory response during infections. It should be emphasized that among those cytokines/chemokines are IFNs taking part in the defense against viral agents. Also, it was found that BD-2 could stimulate a PMC migratory response as high as in the case of other AMPs, i.e., CRAMP, LL-37, and such factors as NGF, TNF, or CCL5, which indicates that these peptides may act as potent chemoattractants, particularly in their milieu. Likewise, in this paper, we documented that BD-2 activates MCs to ROS generation. These findings bring us closer to regarding BD-2 as an active participant in the MC antimicrobial immune response. We also stated that IgE priming amplified PMC degranulation and migratory response upon BD-2 incubation. The observation that IgE-coated MC histamine release is elevated upon MC response to BD-2 might be of great importance due to the known pathobiological role of this mediator in allergy. Furthermore, it can be assumed that in the case of allergies, there is an enhanced MC immune response and cell influx to the site of the ongoing infection.

To date, it has not been entirely clear what are the exact defensin concentrations in physiological and pathological conditions. In healthy individuals, the BD-2 levels were as low as 0.3 pg/mL in bronchoalveolar lavage fluid and 47.6 pg/mL in plasma [36]. In healthy adult individuals, the serum concentration of this peptide is within the range 36–800 pg/mL [37, 38]. Furthermore, levels of BD-2 in the saliva of healthy adults are significantly higher and amount up to 9.5 *μ*g/mL [39]. In our studies conducted *in vitro*, we used BD-2 at 1 *μ*g/mL to assess antimicrobial peptide impact on TLR/RLR expression and concentrations from 1 to 40 *μ*g/mL to determine whether BD-2 stimulation induces the MC histamine/cytokine/chemokine/ROS generation and migratory response. Therefore, we can assume that concentrations of BD-2 applied in our studies reflect the defensins' physiological levels.

## 5. Conclusion

Our findings demonstrate that the expression of TLR3, TLR7, TLR9, and RIG-I is enhanced by treatment with BD-2 in *in vivo* differentiated PMCs. Our observations show that BD-2 may stimulate PMCs to release proinflammatory and immunoregulatory mediators and to induce a migratory response. Taken together, we proved that BD-2 could modulate both the phenotype and the reactivity of tissue MCs. In conclusion, our data highlight that BD-2 might strongly influence MC features and activity, mainly by strengthening their role in the inflammatory mechanisms and controlling the activity of cells participating in antimicrobial processes. Since studied receptors sense viral antigens and the mechanisms studied are involved in antiviral defense, these results are suggesting that defensins may augment the capability of PMCs to detect viral-associated molecular patterns and strengthen the role of those cells in the viral immune response.

## Figures and Tables

**Table 1:** Sequences of primers used in the study.

Gene name	Primer sequence (5′-3′)
*Actb*	Forward: TCTGTGTGGATTGGTGGCTCTA Reverse: CTGCTTGCTGATCCACATCTG

*TLR3*	Forward: GATTGGCAAGTTATTCGTC Reverse: GCGGAGGCTGTTGTAGG

*TLR7*	Forward: GTTTTACGTCTACACAGTAACTCTCTTCA Reverse: TTCCTGGAGGTTGCTCATGTTTT

*TLR9*	Forward: CCGAAGACCTAGTCTA Reverse: TGATCACAGCGACGGCAATT

*IL-1β*	Forward: CACCTCTCAAGCAGAGCACAG Reverse: GGGTTCCATGGTGAAGTCAAC

*IL-4*	Forward: ATGCACCGAGATGTTTGTACC Reverse: TTTCAGTGTTCTGAGCGTGGA

*IL-6*	Forward: TCCTACCCCAACTTCCAATGCTC Reverse: TTGGATGGTCTTGGTCCTTAGCC

*IL-10*	Forward: CACTGCTATGTTGCCTGCTC Reverse: TTCATGGCCTTGTAGACACC

*IL-18*	Forward: AAACCCGCCTGTGTTCGA Reverse: ATCAGTCTGGTCTGGGATTCGT

*IL-33*	Forward: TCGCACCTGTGACTGAAATC Reverse: ACACAGCATGCCACAAACAT

*TNF*	Forward: AAATGGGCTCCCTCTCATCAGTTC Reverse: TCTGCTTGGTGGTTTGCTACGAC

*CCL2*	Forward: ATGCAGTTAATGCCCCACTC Reverse: TTCCTTATTGGGGTCAGCAC

*CCL3*	Forward: CATGGCGCTCTGGAACGAA Reverse: TGCCGTCCATAGGAGAAGCA

*CCL4*	Forward: TATGAGACCAGCAGCCTTTGC Reverse: GCACAGATTTGCCTGCCTTT

*IFN-α*	Forward: CTGCTGTCTAGGATGTGACCTGC Reverse: TTGAGCCTTCTGGATCTGCTG

*IFN-β*	Forward: CGTTCCTGCTGTGCTTCTC Reverse: TGTAACTCTTCTCCATCTGTGAC

*IFN-γ*	Forward: ACGCCGCGTCTTGGTTT Reverse: AGGCTTTCAATGAGTGTGCTT

*GM-CSF*	Forward: AGACCCGCCTGAAGCTATACAA Reverse: CTGGTAGTGGCTGGCTATCATG

*TGF-β*	Forward: CGTGGAAATCAATGGGATCAG Reverse: GGAAGGGTCGGTTCATGTCA

## Data Availability

The data used to support the findings of this study are included within the article.
